# PHGDH at the crossroads: metabolic plasticity, metastatic paradoxes, and therapeutic reconnaissance in cancer

**DOI:** 10.1186/s12929-025-01205-y

**Published:** 2026-01-05

**Authors:** Liang Hao, Bai-Qiang Li, Shi-Yang Lu, Zhong-Cai An, Zheng-Yuan Yin, Zhen-Xian Du, Hua-Qin Wang

**Affiliations:** 1 Department of Biochemistry & Molecular Biology, China Medical University, Shenyang, 110122 China; 2 Key Laboratory of Cell Biology, Ministry of Public Health, and Key Laboratory of Medical Cell Biology, Ministry of Education, China Medical University, Shenyang, 110122 China; 3 Department of Endocrinology & Metabolism, The 1st Affiliated Hospital, China, Medical University, Shenyang, 110001 China; 4 Department of Chemistry, School of Forensic Medicine, China Medical University, Shenyang, 110122 China

**Keywords:** PHGDH, Serine biosynthesis pathway, Neurotoxicity paradox, Metastatic heterogeneity, Precision metabolic oncology, Spatial multi-omics

## Abstract

Phosphoglycerate dehydrogenase (PHGDH), the rate-limiting enzyme of the serine biosynthesis pathway (SSP), is a central metabolic hub and multifunctional oncoprotein that drives tumorigenesis through both canonical and non-canonical mechanisms. This review outlines the multi-level regulation of PHGDH, covering epigenetic remodeling (DNA hypomethylation, H3K4me3/H3K36me3 dynamics), transcriptional control (ATF4, MYC, EWS-FLI1), post-transcriptional fine-tuning (m^6^A/m^5^C modifications, RNA-binding proteins), and post-translational modifications (ubiquitination, methylation, phosphorylation). Together, these regulatory layers allow cancer cells to adapt metabolically to microenvironmental stress. Beyond its fundamental role in supplying nucleotides, maintaining redox homeostasis, and supporting one-carbon metabolism, PHGDH also performs moonlighting function. For example, its translocation to the nucleus inhibits PARP1 to sustain oncogenic transcription, while its presence in mitochondria helps remodel electron transport chains to promote metastasis. Critically, PHGDH exhibits a therapeutic paradox wherein its inhibition can synergize with chemotherapy, radiotherapy, and immunotherapy across diverse malignancies, yet tumors develop resistance via metabolic plasticity, or by selection of PHGDH-low metastatic clones. The clinical translation of PHGDH inhibitors is further challenged by inherent neurotoxicity risks, as neurons rely on de novo serine synthesis. To address these challenges, we propose a precision roadmap that integrates spatial multi-omics, AI-driven allosteric inhibitor design, dynamic biosensing (e.g., ^18^F-metabolite PET), and biomarker-stratified clinical trials. By reconciling the dual nature of PHGDH biology, we can transform this metabolic linchpin from a confounding paradox into a clinically actionable vulnerability.

##  PHGDH as the metabolic fulcrum in oncogenesis and therapeutic vulnerability

Cancer metabolism is now a cornerstone of oncogenesis research. The serine biosynthesis pathway (SSP) is a metabolic linchpin for tumor survival, proliferation, and therapeutic resistance. At its center is phosphoglycerate dehydrogenase (PHGDH), the rate-limiting enzyme that diverts glycolytic flux toward de novo serine synthesis. Once considered a simple housekeeping catalyst, PHGDH is now recognized as a master regulatory oncoprotein. It is frequently upregulated across cancers to drive anabolic demands, maintain redox balance, facilitate epigenetic reprogramming, and mediate microenvironmental crosstalk[1, 2].

This review examines how PHGDH is controlled at multiple levels, including epigenetic remodeling, transcriptional control, post-transcriptional fine-tuning, and post-translational modifications. Collectively, these mechanisms enable adaptive metabolic fitness under stress. Cancer-specific contexts, dictated by oncogenic drivers (e.g., *MYC* [3, 4], EWS-FLI1 [5, 6]), tumor suppressors (e.g., Parkin [7]), and microenvironmental insults, dynamically tune PHGDH expression and activity. For instance, DNA hypomethylation enables *PHGDH* induction in nutrient-starved pancreatic tumors [8], while ATF4 integrates endoplasmic reticulum stress, hypoxia, and oncogenic signals to co-activate SSP genes including *PHGDH* [9, 10]. Beyond its canonical metabolic role, PHGDH performs non-canonical roles. Under glucose scarcity, PHGDH translocates to the nucleus and inhibits PARP1, thereby perpetuating oncogenic transcription [11]. Meanwhile, mitochondrial PHGDH forms complex with ANT2 to reconfigure respiratory complexes and potentiate metastatic dissemination [12].

PHGDH represents a therapeutic double-edged sword. Its inhibition synergizes with conventional chemotherapy (e.g., resensitizing FLT3-ITD⁺ AML to cytarabine [10]) and emerging immunotherapies (e.g., enhancing CAR-T infiltration in glioblastoma by normalizing vasculature [13]). However, tumors deploy evolutionary countermeasures, such as metabolic plasticity [14] or selective expansion of PHGDH-low metastatic subclones [15], demonstrating the resilience of serine metabolism. Compounding this challenge is the risk of neurotoxicity, arising from neuronal dependence on de novo serine synthesis [16]. This creates a conflict with the finding that brain metastases can exploit PHGDH to bypass blood–brain barrier (BBB) serine restrictions [17].

To navigate these challenges, we chart a precision roadmap that integrates artificial intelligence for designing allosteric inhibitors targeting cryptic structural pockets [18], spatial multi-omics to decode microenvironmental metabolic heterogeneity [19, 20], and biomarker-stratified clinical trials incorporating metabolic imaging (e.g., ^18^F-FGln PET [21]). By reconciling the dual nature of PHGDH biology and balancing antitumor efficacy against neurotoxicity risks and metastatic trade-offs, we aim to transform this metabolic fulcrum from a biological paradox to a therapeutically actionable vulnerability.

## Hierarchical control networks dictating PHGDH dysregulation in malignancies

**Fig. 1:**
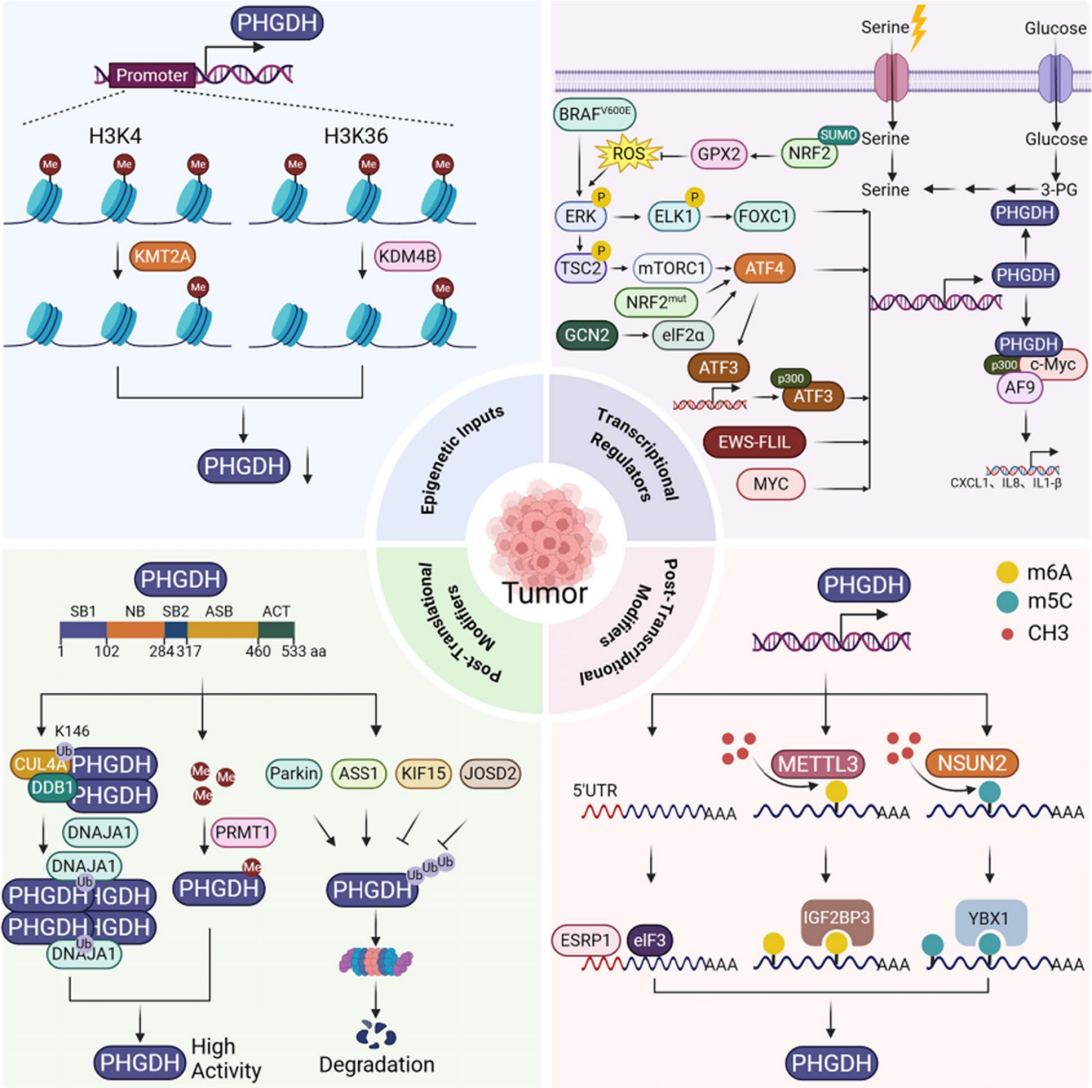
Hierarchical regulation network of PHGDH in cancer

As the rate-limiting enzyme of de novo serine biosynthesis, PHGDH is a pivotal metabolic node frequently dysregulated in cancers. Pathological PHGDH overexpression fuels nucleotide synthesis, redox equilibrium, and one-carbon flux, driving oncogenic progression and therapeutic resistance. This section describes the multilayered regulatory networks controlling PHGDH across epigenetic, transcriptional, post-transcriptional, and post-translational levels (Fig. 1). These layers illustrate how tumors exploit layered control mechanisms to achieve metabolic adaptability.

The schematic illustrates the multi-layered control of PHGDH across four regulatory tiers in cancer.

*Epigenetic Regulation:* Histone modifications mediated by KMT2A (activation) and KDM4B (repression) modulate *PHGDH* promoter accessibility.

*Transcriptional Regulation:* Transcription factors (ATF3, ATF4, EWS-FLIL, FOXC1, MYC), oncogenic signals (BRAF^V600E^, mTORC1, ERK), and stress (ROS) pathways drive *PHGDH* gene expression.

*Post-Transcriptional Regulation:* RNA-binding proteins (elF3, YBX1, IGF2BP3), and mRNA translational regulators (METTL3, NSUN2) control *PHGDH* mRNA stability, translation, and abundance.

*Post-Translational Modifications (PTMs):* Ubiquitination (mediated by ASS1, KIF15, Parkin, JOSD2, CUL4A-DDB1) and methylation (mediated by PRMT1) regulate PHGDH protein stability, degradation, and enzymatic activity.

### Epigenetic determinants of PHGDH accessibility

Neoplasm-specific chromatin dynamics shape PHGDH expression through coordinated DNA and histone modifications. Promoter hypomethylation is a key activation switch. In pancreatic ductal adenocarcinoma (PDAC), this epigenetic shift licenses *PHGDH* induction under nutrient stress [8], while bladder carcinogenesis couples hypomethylation with genomic amplification to enforce pathological overexpression [22]. Histone modifications further refine transcriptional control. KDM4B-mediated H3K36me3 erasure suppresses *PHGDH* activity [23], whereas Menin-MLL/TrxG complexes deposit activating H3K4me3 in Ewing sarcoma to prime SSP genes for nucleotide biosynthesis [24]. Critically, these epigenetic alterations modulate transcriptional kinetics by reorganizing enhancer-promoter architectures and modulating RNA polymerase II processivity during metabolic perturbation.

### Transcriptional circuits enabling context-dependent expression

A hierarchy of stress-responsive transcription factors converges on *PHGDH* regulation. ATF4 functions as the master stress integrator, induced via the GCN2-eIF2α axis by nutrient deprivation, hypoxia, ER stress, or oncogenic signaling [9, 10]. Beyond direct *PHGDH* transactivation, ATF4 stabilizes the SSP network through ATF3 induction. This creates a feedforward loop amplified by p300 co-activation of *PHGDH*, *PSAT1*, and *PSPH* expression to sustain survival under metabolic constraint [9]. This axis coordinates adaptive responses via amino acid transporters (e.g., SLC1A5) and one-carbon enzymes (e.g., SHMT2, MTHFD2), reinforcing nucleotide synthesis, redox homeostasis, and mTORC1 activation in T-ALL [25].

This stress-responsive logic extends to transcriptional regulators. For instance, FOXC1 in CRC calibrates SSP activity to microenvironmental serine availability rather than constitutively reprogramming basal metabolism [26]. Similarly, SUMOylation-potentiated NRF2 (K110) activates PHGDH to sustain redox balance in HCC [27]. Functional indispensability is underscored by tumor suppression upon PHGDH ablation in melanoma and AML [10, 28]. The ER stress sensor ERN1 (IRE1α) fine-tuning this circuity in glioblastoma [29, 30].

Lineage-specific drivers impose additional complexity. The Menin-MLL complex directly occupies the *PHGDH* promoter in Ewing sarcoma, diverting glycolytic flux toward serine/glycine biosynthesis [24]. This creates an NCT-503-targetable vulnerability [31]. Similarly, MYC-family oncoproteins directly occupy the *PHGDH* promoter, driving overexpression in Group 3 medulloblastoma and MYCN-amplified neuroblastoma [3, 4]. This enhances glucose-derived serine flux, sustaining nucleotide pools while creating therapeutically exploitable vulnerabilities [3, 4]. MYC further intersects with broader networks to regulate PHGDH expression. In glioblastoma stem-like cells (GSCs), it activates PHGDH to confer radioresistance via redox modulation [32], while wild-type IDH1 stabilizes PHGDH to preserve NSCLC stemness [33]. Nuclear PHGDH also collaborates with MYC to transactivate immunosuppressive chemokines (CXCL1/IL8), thereby remodeling the HCC microenvironment [34].

Therapeutic exploitation reveals context-dependent synergies. ATF4-PHGDH targeting sensitizes FLT3-ITD⁺ AML to cytarabine [10], overcomes FGFR1/glutaminase inhibitor resistance [25], and normalizes vasculature to potentiate CAR-T efficacy in glioblastoma [13]. Similarly, MYC-PHGDH ablation restores radiosensitivity in GSCs [32], while PHGDH inhibitors combined with mTORC1 blockade induce osteosarcoma apoptosis [35]. Notably, partial serine reduction (in vivo diet + inhibitors) achieves efficacy without complete pathway ablation, suggesting clinically feasible thresholds[36].

### Real-time tuning via post-transcriptional regulation

Post-transcriptional mechanisms enable rapid adaptation through RNA modifications and binding proteins. METTL3-mediated m^6^A methylation stabilizes *PHGDH* transcripts via IGF2BP3 recognition in HCC and AML [37, 38], while NSUN2-deposited m^5^C reinforces stability in AML, establishing a dual-modification amplification system [39]. RNA-binding proteins exert complementary control. ESRP1 binding to the *PHGDH* 5′-UTR enhances transcript stability in therapy-resistant ER^+^ breast cancer [40, 41], whereas eIF3i recognizes m^6^A marks to selectively promote PHGDH translation in colorectal cancer [42]. Collectively, these mechanisms permit dynamic recalibration of PHGDH output to meet metabolic demands.

### Post-translational switches dictating functional plasticity

Post-translational modifications (PTMs) serve as molecular rheostats, regulating PHGDH stability, subcellular localization, and catalytic activity. Ubiquitination dynamically modulates protein turnover. Parkin destabilizes PHGDH via K330 ubiquitination [7], whereas Josephin-2-mediated deubiquitylation stabilizes it to promote stemness in HCC [43]. Crucially, Cul4A-DDB1-mediated monoubiquitylation at K146 enhances enzymatic activity by stabilizing the catalytically competent tetramer, thereby amplifying S-adenosylmethionine (SAM) production for epigenetic reprogramming [44].

Diverse PTMs directly reconfigure PHGDH function. PRMT1-mediated methylation activates PHGDH in HCC [45, 46] and drives resistance in TNBC [20]; p38-dependent phosphorylation at S371 triggers nuclear translocation during glucose restriction [11]; and AMPK-mediated phosphorylation at S55 redirects flux toward malate oxidation, generating nuclear NADH to support epigenetic remodeling [11]. Protein interactors also modulate outcomes. ASS1 binding promotes ubiquitin-mediated degradation in TNBC [47], while KIF15 stabilizes PHGDH to maintain ROS homeostasis in HCC stem cells [48]. These multilayered modifications establish PHGDH as a stress-responsive signaling hub that interprets metabolic crises through covalent switching.

The repertoire of PTMs regulating PHGDH plasticity continues to expand. A recent study reveals a novel regulatory axis involving the cell cycle kinase PLK1, which is frequently overexpressed in advanced prostate cancer. PLK1 directly phosphorylates PHGDH at three specific serine residues (S512, S513, S517) [49]. This multi-site phosphorylation reduces PHGDH protein levels and enzymatic activity. Consequently, PLK1-mediatedinhibition of the SSP forces cancer cells to rely critically on exogenous serine uptake via the ASCT2 transporter. This metabolic shift fuels the biosynthesis of lipids, including sphingolipids, which are essential for tumor growth and survival in this context. This mechanism links a master regulator of cell division directly to serine metabolism reprogramming, suggesting that therapeutic targeting of the SSP, serine uptake, or downstream lipid synthesis may be particularly relevant in PLK1-high advanced cancers.

### PHGDH as a metabolic stress integrator

PHGDH functions as a critical stress sensor, upregulated by diverse insults to preserve cellular fitness. Serine/glycine deprivation activates a multi-transcription factor response involving ATF4 [10], FOXC1 [26], and cooperation between BRAF^V600E^ and ATF4 in melanoma [28] to induce PHGDH expression. Glutamine restriction in gastric cancer triggers ATF4/CEBPB-driven *PHGDH* upregulation to sustain nucleotide synthesis and redox homeostasis [50]. Glucose restriction triggers AMPK/p38-dependent phosphorylation, inducing PHGDH nuclear translocation to sustain tumor growth under nutrient stress [11]. Oxygen deprivation upregulates PHGDH expression in glioblastoma, while ERN1 knockdown enhances this hypoxic induction by releasing repression [29, 30]. This adaptive plasticity establishes PHGDH as a pleiotropic metabolic hub that deciphers environmental challenges through context-specific circuits, enabling tumor survival under nutrient stress.

## PHGDH as a multifunctional orchestrator of tumor fitness and therapeutic resistance

The oncogenic role of PHGDH extends far beyond its canonical catalytic function in serine biosynthesis. As comprehensively reviewed by Wang et al., PHGDH exhibits a wide range of non-canonical or 'moonlighting' functions that significantly alter our understanding of how it regulates tumor cell fate [51]. In addition to its metabolic role, PHGDH participates in regulating gene transcription and translation, potentially offering new therapeutic avenues. The following sections delineate both the core metabolic functions and these multifaceted non-canonical networks through which PHGDH orchestrates tumor progression and therapy resistance.

### Core metabolic functions and signaling integration

**Fig. 2:**
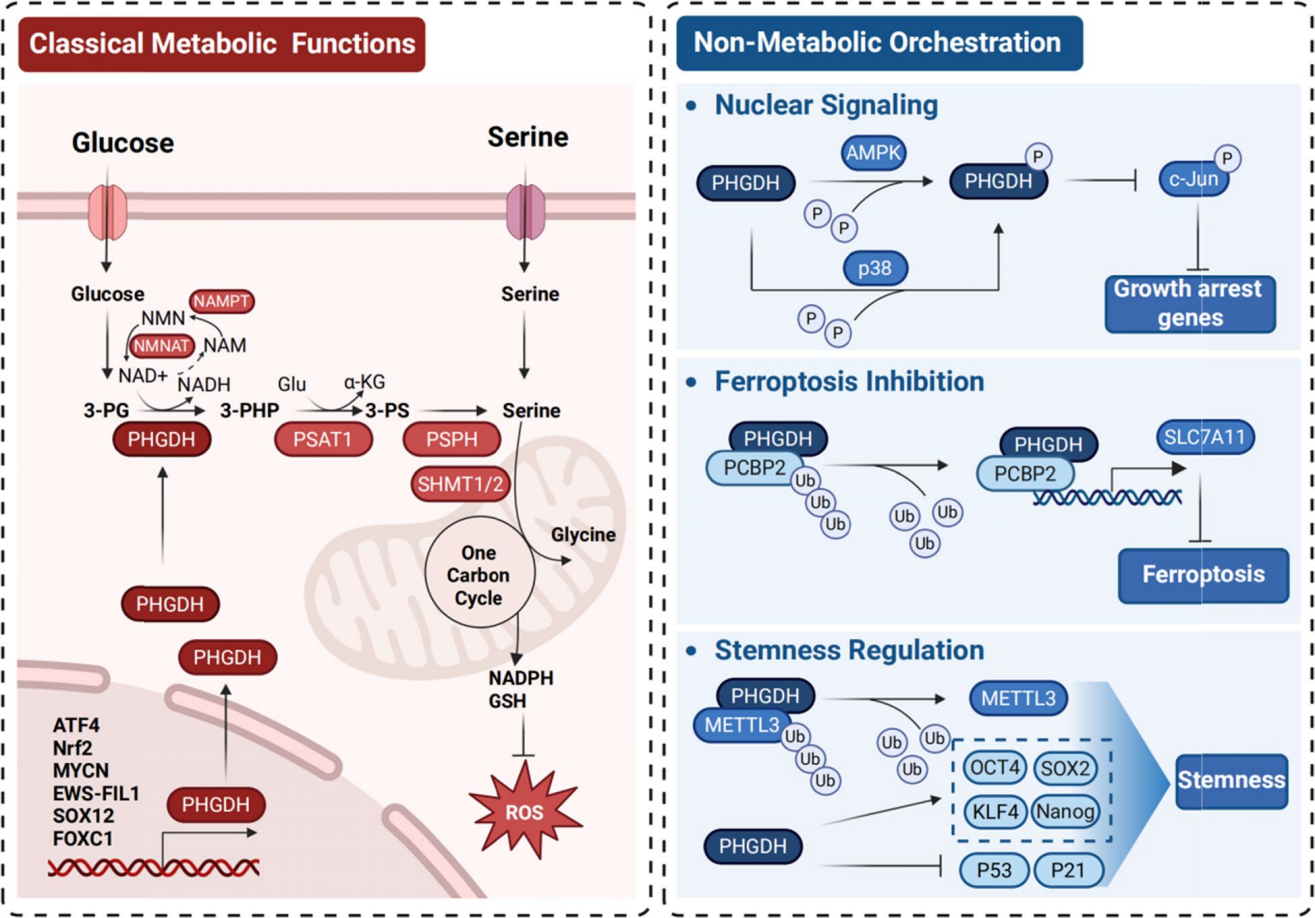
Metabolic and non-canonical functions of PHGDH drive oncogenesis

As the rate-limiting enzyme of serine biosynthesis, PHGDH serves as a central metabolic orchestrator in malignancies (Fig. 2). By diverting glycolytic 3-phosphoglycerate toward de novo serine production, it sustains two critical anabolic processes: providing precursor for purine/pyrimidine nucleotide synthesis [52–54], and generating one-carbon units essential for histone/DNA methyltransferase-mediated epigenetic reprogramming [26, 53]. Concurrently, pathway activation yields NADPH, a key cofactor that synergizes with glutathione (GSH) to neutralize therapy-induced reactive oxygen species (ROS), thereby conferring resistance to chemo- and radiotherapy in glioblastoma, neuroblastoma, and multiple myeloma [5, 32, 37, 55–57].

PHGDH further enables metabolic self-sufficiency under nutrient constraint. In MYCN-amplified neuroblastoma, endogenous serine biosynthesis circumvents dependency on exogenous serine/glycine, preventing nucleotide pool collapse during deprivation [4]. Critically, PHGDH inhibition induces compensatory adaptations, including enhanced amino acid transport and lipogenesis, that facilitate metabolic escape [58], underscoring the need for combinatorial strategies. Preclinical targeting demonstrates that NCT-503 suppresses Ewing sarcoma progression [31], while genetic ablation confirms PHGDH as a survival dependency in neuroblastoma [59, 60]. To circumvent resistance, rational combinations, such as dual NAD⁺ pathway inhibition or dietary serine restriction, can fully exploit this vulnerability [31, 36, 54].

The functional impact of PHGDH extends beyond the tumor cell itself to critically shape the metastatic niche. Deletion or pharmacological inhibition of PHGDH in osteoclast progenitors impaired their differentiation and increased bone mass, thereby preventing bone loss in a mouse model of postmenopausal osteoporosis [61]. This indicates that transient activation of the SSP is essential for osteoclast differentiation. Mechanistically, SSP-derived α-ketoglutarate serves as a necessary cofactor for histone demethylases that remove repressive marks at the *Nfatc1* locus, inducing NFATc1 expression and subsequent osteoclast maturation. This metabolic-epigenetic coupling reveals how PHGDH activity within the bone microenvironment directly facilitates osteoclastogenesis, providing a mechanism for the recognized dependency of bone metastasis on PHGDH[62] and highlighting a non-cell-autonomous role for PHGDH in promoting pathological bone resorption.

### Non-canonical contextual-dependent oncogenic networks

PHGDH functions as a multidimensional signaling hub where non-canonical activities and tissue-specific dependencies converge through spatially organized interactomes and PTMs, driving context-dependent tumor progression, stemness, and therapy resistance (Fig. 2).

PHGDH promotes tumor progression through its classical metabolic functions and non-canonical signaling roles.

*Canonical Metabolic Functions:* PHGDH catalyzes the first step in de novo serine biosynthesis from glucose-derived 3-phosphoglycerate (3-PG), generating 3-phosphohydroxypyruvate (3-PHP). Subsequent enzymes PSAT1 and PSPH complete serine synthesis. Serine fuels one-carbon metabolism via SHMT1/2, producing glycine, NADPH, and precursors for glutathione (GSH) synthesis, thereby maintaining redox balance and scavenging ROS. Serine-derived flux also supports NAD^+^ regeneration via the NMNAT/NAMPT salvage pathway.

*Non-Canonical Signaling Functions:* PHGDH phosphorylation by AMPK (Ser55) and p38 (Ser371) regulates its subcellular localization and non-enzymatic activities. Nuclear PHGDH activates c-Jun and suppresses growth arrest genes. PHGDH interaction with PCBP2 stabilizes SLC7A11, inhibiting ferroptosis. PHGDH promotes stemness by upregulating pluripotency factors (OCT4, SOX2, KLF4, NANOG) and downregulating p53/p21.

### Subcellular localization dictates functional output

Nuclear translocation under glucose scarcity (Sect. "PHGDH as a Metabolic Stress Integrator") exemplifies PHGDH’s functional plasticity, repurposing it from a metabolic catalyst to a transcriptional co-regulator [11]. In the nucleus, it cooperates with MYC to transactivate immunosuppressive chemokines (CXCL1/IL8), remodeling the HCC microenvironment [34]. Separately, mitochondrial PHGDH-ANT2 complexes accelerate respiratory chain biogenesis via mtEFG2 recruitment, directly potentiating metastasis [12]. Strikingly, expression heterogeneity generates paradoxical phenotypes. PHGDH-low states enhance αvβ₃-integrin sialylation through HEXA-HEXB axis activation, while mitochondrial PHGDH promotes invasion via oxidative phosphorylation remodelin g[15].

#### PTM-epigenetic crosstalk drives pathological reprograming

Cul4A-DDB1-mediated monoubiquitylation at Lys146 elevates SAM production, inducing SETD1A-dependent H3K4me3 modifications that upregulate metastasis-associated genes (e.g., *LAMC2*, *CYR61*) in colorectal cancer [44]. Conversely, PRMT1-mediated arginine methylation (R54/R20) activates PHGDH to stimulate FASN-dependent palmitate production, establishing chemoresistant lipid barriers via S-palmitoylation feedback loops [20]. Reciprocally, epigenetic regulators modulate PHGDH. KDM4B deficiency enriches promoter H3K36me3 to sustain breast cancer stemness [23], while SUMOylated NRF2 (K110) transcriptionally activates *PHGDH* to bolster antioxidant defenses in HCC [27]. Non-catalytic functions further expand its role, exemplified by PHGDH-mediated upregulation of pluripotency factors (OCT4/SOX2/NANOG) in thyroid malignancies [63].

#### Lineage-specific interactomes define therapeutic vulnerabilities

Context-dependent protein interactions execute tissue-specific programs. In bladder cancer, PHGDH stabilizes PCBP2 to inhibit SLC7A11 ubiquitination, suppressing ferroptosis and conferring chemoresistance [57]. In HCC, PHGDH binding to METTL3 drives epithelial-mesenchymal transition (EMT) [64]. Conversely, ASS1 interaction triggers PHGDH self-ubiquitination and degradation, suppressing tumorigenesis in TNBC [47]. TNBC alternatively relies on a self-reinforcing PRMT1-PHGDH-FASN axis targetable by FASN inhibitors (e.g., TVB-2640) [20]. Scaffolding functions include eIF4A1/eIF4E binding in pancreatic cancer to enhance oncoprotein translation [65], while the circMYBL2-encoded p185 promotes UCHL3-mediated PHGDH degradation to inhibit colorectal cancer invasion [66].

#### Stemness-differentiation equilibrium regulation

PHGDH bidirectionally regulates cellular identity through tissue-specific mechanisms. It enforces stemness by upregulating pluripotency factors (OCT4/SOX2/KLF4) in thyroid cancer [63] and sustains AML stemness via ATP-P2X7 signaling [67]. Conversely, its ablation sensitizes NSCLC stem cells to gemcitabine by disrupting self-renewal [33]. This functional plasticity establishes PHGDH as a dynamic rheostat of pluripotency-differentiation balance, modulated by microenvironmental metabolites and regulatory networks.

#### The PHGDH-NAD^+^ axis: a metabolic nexus in cancer and immunity

The interplay between PHGDH and nicotinamide adenine dinucleotide (NAD⁺) metabolism is a pivotal axis in cellular adaptation, with profound implications in cancer and immunity. PHGDH-driven serine biosynthesis depends on NAD⁺ as a cofactor, creating a metabolic nexus influencing redox balance, nucleotide synthesis, and cell survival under stress. For instance, in glioblastoma, PHGDH supports hypoxia tolerance by maintaining NADPH levels and redox homeostasis, mitigating ROS-induced death [55]. Similarly, the NAD⁺ salvage pathway is essential for sustaining PHGDH activity in breast cancers, where NAMPT-mediated NAD⁺ regeneration promotes serine production and tumor growth [54]. This dependency is exploited therapeutically, as PHGDH inhibition synergizes with NAMPT inhibition in Ewing sarcoma and with tyrosine kinase inhibitors in HCC to overcome chemoresistance [31, 68].

Beyond cancer, this metabolic crosstalk extends to immune regulation. A study by Wang et al. reveals that in inflammatory macrophages, PHGDH-derived serine synthesis restricts NAD⁺ accumulation, thereby inhibiting SIRT1/SIRT3 activity. This leads to heightened acetylation of histones (e.g., H3K9/27) and inflammasome components (e.g., NLRP3 and ASC), amplifying IL-1β production and exacerbating systemic inflammation [69]. This mechanism underscores a non-canonical role for PHGDH in linking serine metabolism to NAD⁺-dependent epigenetic and post-translational control of immunity.

Moreover, nutrient stress rewires PHGDH-NAD⁺ dynamics to foster metabolic plasticity. Under glutamine deprivation, gastric cancer cells upregulate PHGDH to sustain mitochondrial folate cycling and NADPH generation, promoting survival [50]. Conversely, in ovarian cancer, platinum resistance is associated with reduced PHGDH expression, which enhances NAD⁺ regeneration to support PARP-mediated DNA repair [70]. These findings illuminate PHGDH as a metabolic linchpin whose interplay with NAD⁺ governs diverse pathophysiological outcomes, offering novel targets for therapy in oncology and inflammatory diseases.

##  Therapeutic landscape and translational challenges in targeting PHGDH

### Mechanism-guided inhibitor development

The strategic targeting of PHGDH has evolved beyond catalytic site inhibition (Table 1). Direct inhibitors such as NCT-503 (targeting the NAD⁺-binding pocket) [59] demonstrate multifaceted efficacy. They suppress pulmonary neoplasia progression, sensitize cerebral malignancies to temozolomide through MGMT downregulation, and reduce stemness markers in thyroid carcinomas [59, 63, 71]. Analogously, CBR-5884 activates ROS/Wnt/β-catenin cascades, potentiating olaparib response in ovarian cancer [72]. Natural compounds provide distinct chemotypes. Lithospermic acid C targets the cofactor-binding domain [73], homoharringtonine disrupts enzymatic function [60], and Iox A acts as an allosteric inhibitor [74].

**Table 1:** PHGDH inhibitors: mechanisms efficacy, and clinical status

Inhibitor	Class/target	IC50/EC50	BBB permeability	Model SystemTested	Key preclinical findings	Resistance mechanisms	Clinical status	Refs
NCT-503	Competitive NAD^+^ binding site inhibitor	2.8 μM	Low	Ewing sarcomaxenografts Glioblastoma models Thyroid cancer cell lines Ovarian cancer models	Suppresses Ewing sarcoma growth (decrease tumor volume 70%) Sensitizes glioblastoma to TMZ (decrease MGMT expression) Blocks stemness markers in thyroid cancer	SLC1A4-mediated serine uptake mTORC1-driven lipogenesis	Preclinical	[31, 59, 63, 71]
CBR-5884	Allosteric inhibitor	~ 5 μM	Moderate	Medulloblastoma models	Induces ROS/Wnt/β-catenin in ovarian cancer Synergizes with olaparib (decrease tumor growth 60%) Extends survival in medulloblastoma models	ATF4/CEBPB axis activation HK2/GLUD1 rewiring	Preclinical	[72]
Homoharringtonine	Moderate Natural alkaloid(PHGDH disruptor)	0.7 μM	High (Risk)	High-risk neuroblastoma models Cerebral metastasis models	Depletes cerebral serine neurotoxicity Effective in high-risk neuroblastoma (increase apoptosis)	Josephin-2-mediated stabilization	Preclinical	[60]
Lithospermic acid C	Natural compound (cofactor domain)	15.3 μM	ND	Computational models in vitro enzyme assays	Binds cofactor domain in computational models Reduces SAM production	ND	Computational	[73]
lox A	Tomatillo-derived allosteric inhibitor	9.8 μM	Low	PDAC xenografts Pancreatic cancer cell lines	Suppresses PDAC growth (decrease xenograft 50%) Disrupts elF4A1 interaction	elF3i-mediated translational escape	Preclinical	[74]
PKUMDL-WQ-2201	Catalytic site inhibitor	> 10 μM	ND	NSCLC models Various cancer cell lines	Synergizes with gemcitabine in NSCLC Limited potency and off-target effects	Parkin deficiency → impaired degradatiol	Preclinical	[81]
D8 derivatives	BBB-restricted analogs	0.3–1.2 μM	Negligible	Peripheral tumormodels CNS-sparingefficacy models	Designed to spareCNS: no neuronal serine depletion Maintains peripheral antitumor efficacy	N/A		[59]

Indirect strategies exploit upstream vulnerabilities. Epigenetic modulators such as the METTL3 inhibitor STM2457 degrade *PHGDH* transcripts [37], while transcriptional suppression of NAT10 impedes cerebral metastatic adaptation [17]. Targeting PHGDH-activating PTMs, exemplified by FBXO7-mediated PRMT1 degradation [45], represents an emerging therapeutic axis.

### Rational combinatorial approaches against resistance

Although survival extension in xenograft models supports target engagement [4], PHGDH inhibition in MYCN-amplified neuroblastoma models primarily induces cytostatic effects rather than cytotoxicity, and tumors rapidly develop resistance through metabolic rewiring [4]. To circumvent adaptive resistance, dual metabolic targeting combines PHGDH inhibitors (e.g., NCT-503) with NAMPT antagonists to deplete NAD⁺ pools, inducing synthetic lethality [31, 54]. Dietary modulation strategies leverage serine/glycine restriction concomitant with CBR-5884 administration. This provokes toxic deoxysphingolipid accumulation that elicits lethal metabolic disruption [36, 75].

PHGDH inhibitors further restore sensitivity to molecularly targeted agents. They can reverse EGFR inhibitor resistance in lung cancer [76], MEK inhibitor refractoriness in melanoma [77], sorafenib resistance in HCC [68], and BRAF inhibitor failure across diverse cancers [28]. Additionally, PHGDH blockade enhances immunotherapeutic efficacy; in glioblastoma, it normalizes pathological vasculature to potentiate CAR-T infiltration and cytotoxicity [13].

### Resistance evolution and metabolic adaptation

Tumors evade PHGDH inhibition through metabolic rewiring. They compensate through ATF4/CEBPB-driven reconstitution of NADPH antioxidant systems [50]. mTORC1-dependent lipid remodeling further sustains membrane integrity via monounsaturated fatty acid accumulation [14, 35]. RFWD3-driven PHGDH degradation redirects flux toward NAD⁺/TCA cycle-dependent nucleotide synthesis in osteosarcoma [78]. Genomic adaptations (e.g., SF3B1 mutations) constitutively activate ATF4-driven one-carbon metabolism [79], necessitating combinatorial strategies co-targeting PHGDH and resistance axes.

Chronic evolutionary escapes involve genomic and microenvironmental reprogramming. Genomic alterations stabilize PHGDH through Parkin deficiency (impairing ubiquitin-mediated degradation) [7] or K58 acetylation (inhibiting proteasomal turnover) [80]. Transcriptional reprogramming exploits SF3B1 spliceosome mutations to constitutively activate ATF4-driven one-carbon metabolism [79]. Microenvironmentally, endothelial PHGDH activation induces glycolysis-driven pathological angiogenesis, establishing physical barriers that impede CAR-T infiltration and compromise immunotherapy [13].

### Translational impediments and neurotoxicity constraints

Critical pharmacological limitations hinder clinical translation. Current lead compounds (e.g., NCT-503, PKUMDL-WQ-2201) exhibit suboptimal potency (IC₅₀ > 10 μM) and significant off-target effects[81]. The inherent neurotoxicity constraint presents fundamental challenges. While blood–brain barrier (BBB) penetration is essential for glioma targeting, it risks oligodendrocyte damage and demyelination via central serine depletion[16]. This necessitates compartmentalized therapeutic strategies: BBB-permeable agents for CNS malignancies versus peripherally-restricted compounds for systemic tumors. This represents an unresolved frontier in PHGDH therapeutics.

## Context-dependent duality of PHGDH in metastasis, neurotoxicity, and stemness regulation

### Metabolic determinants of metastatic plasticity

**Fig. 3:**
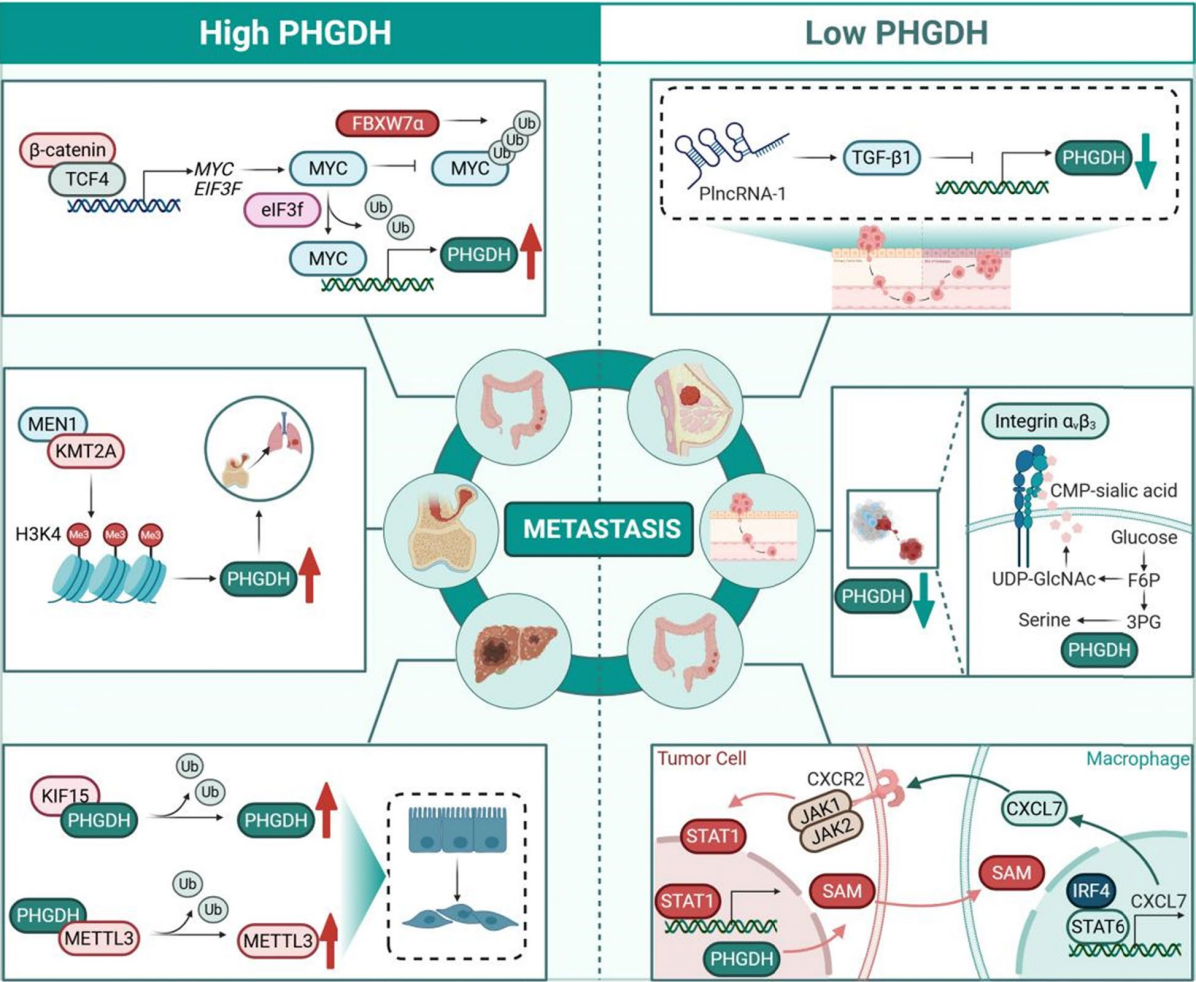
Context-dependent dual roles of PHGDH in tumor metastasis

PHGDH exhibits a context-dependent duality in metastasis regulation, functioning as either a promoter or suppressor of dissemination depending on microenvironmental metabolic constraints (Fig. 3). In nutrient-replete niches of primary tumors and established metastatic sites (e.g., bone, brain), elevated PHGDH drives progression through nucleotide synthesis [2], NADPH/GSH-mediated redox homeostasis [50, 55], and one-carbon unit provision for epigenetic reprogramming [44]. Clinically, high PHGDH correlates with poor prognosis in TNBC, PDAC, and HCC [48, 82–84].

PHGDH expression levels exhibit paradoxical effects on metastasis, influenced by cellular context.

*Pro-Metastatic Functions of High PHGDH Expression:* Transcriptional upregulation by MYC in colorectal cancer. KMT2A-mediated H3K4 methylation promoting PHGDH expression and lung metastasis in osteosarcoma. Interaction with KIF15 stabilizing PHGDH and promoting EMT.

*Pro-Metastatic Functions of Low PHGDH Expression:* TGF-β1 inhibition downregulating PHGDH yet promoting metastasis in breast cancer. Glucose-derived serine pathway supporting UDP-GlcNAc/CMP-sialic acid production for αvβ3 integrin hypersialylation, enhancing invasion in PHGDH-low cells. Macrophage-derived CXCL7 activating CXCR2-STAT1 signaling in tumor cells, leading to SAM production and IRF4/STAT6-mediated polarization of macrophages towards pro-metastatic phenotypes.

Conversely, during circulatory transit or within dormant micrometastases, PHGDH-low clones exhibit enhanced fitness through several mechanisms: αvβ₃-integrin hypersialylation via HEXA-HEXB axis activation [15], reduced anabolic demands enabling quiescence [85], and metabolic plasticity utilizing alternative carbon sources [86]. This manifests as PHGDH-low dominance in circulating tumor populations [15], illustrating evolutionary selection under spatial metabolic constraints. While lung/brain metastases require serine synthesis for mTORC1 activation [87] and BBB adaptation [17], circulating cells exploit PHGDH-low states for dissemination fitness[15, 85]. Consequently, therapeutic PHGDH inhibition risks selecting pre-adapted metastatic clones [15].

### Molecular basis of metastatic organotropism

Tissue-specific contexts dictate the functional role of PHGDH in metastatic progression. Bone metastasis necessitates PHGDH-dependent osteoclast activation [62], while cerebral metastasis exploits PHGDH via NAT10-mediated transcriptional upregulation to circumvent BBB-imposed serine constraints [17]. Molecularly distinct breast cancer subtypes exhibit divergent dependencies. TNBC demonstrate high PHGDH reliance [83], contrasting fundamentally with luminal subtypes where PSAT1 silencing establishes serine auxotrophy [88]. Furthermore, PTMs, including ubiquitination mediated by Parkin or RNF5, coupled with acetylation, dynamically modulate PHGDH abundance, stability, and functional output in response to microenvironmental stress signals [80], thereby fine-tuning its context-dependent contribution to metastatic dissemination.

**Fig. 4:**
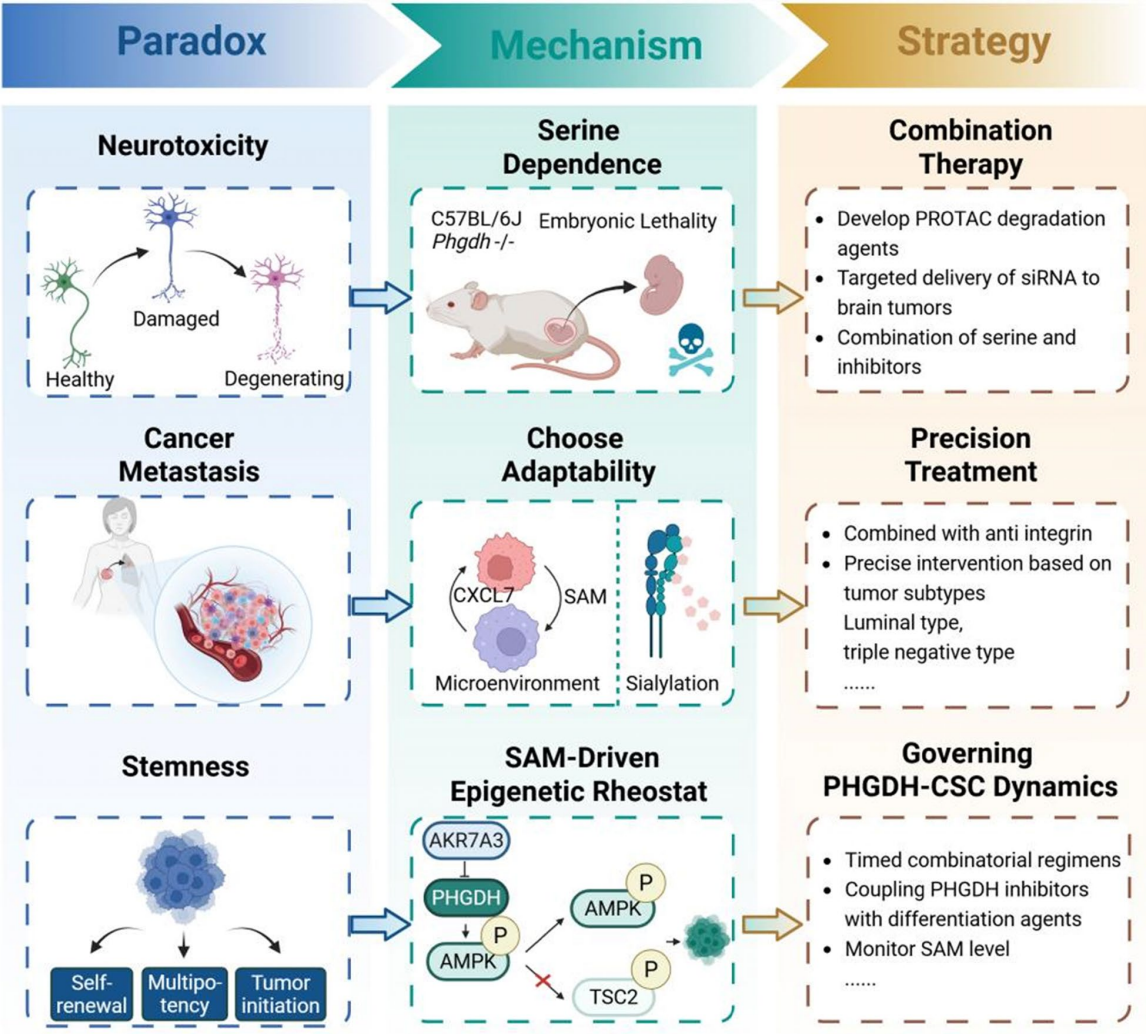
Therapeutic paradoxes of PHGDH inhibition and resolution strategies

Therapeutic precision thus necessitates combinatorial regimens integrating chemotherapy [89] or anti-sialylation agents [15], complemented by subtype-specific metabolic targeting [90] and spatial multi-omics mapping of metastatic niche metabolism [19] (Fig. 4).

Targeting PHGDH in cancer therapy reveals key paradoxes and corresponding resolution strategies.

*Neurotoxicity Paradox:* Systemic PHGDH inhibition depletes serine, causing neuronal damage and degeneration.

Resolution: Employ brain-targeted delivery (e.g., siRNA nanoparticles) or BBB-sparing PROTAC degraders to minimize off-tissue toxicity.

*Metastasis Paradox:* PHGDH suppression may inadvertently promote metastasis via integrin αvβ3 hypersialylation and SAM-driven epigenetic rewiring.

Resolution: Combine PHGDH inhibitors with anti-integrin agents or microenvironment modulators.

*Stemness Paradox:* While serine is essential (evident in PHGDH-null models), tumors maintain stemness via metabolic adaptability despite inhibition.

Resolution: Couple PHGDH inhibitors with differentiation agents (e.g., retinoic acid) and monitor SAM levels to disrupt cancer stemness.

### Neurotoxicity pathophysiology and mitigation

Systemic PHGDH inhibition incurs multifactorial neurotoxicity. Depletion of neuroactive amino acids (glycine/D-serine) impairs synaptic transmission and neuronal excitability [91]. Meanwhile, sphingolipid metabolic derangement precipitates toxic 1-deoxysphinganine accumulation with concomitant sphingosine kinase 1 degradation, disrupting membrane integrity and signaling [92]. Human pathophysiological evidence comes from biallelic PHGDH loss-of-function mutations causing lethal Neu-Laxova syndrome, a neurodevelopmental disorder characterized by severe neurological deficits [93]. This is mechanistically validated in murine models demonstrating demyelination, neurodevelopmental arrest, and motor dysfunction upon neuronal PHGDH ablation [94].

Two complementary mitigation paradigms circumvent neurotoxicity. Biochemical rescue through adjunctive L-serine co-administration preserves neuronal integrity by bypassing de novo serine synthesis requirements. This achieves complete neuroprotection without compromising antitumor efficacy in preclinical models [36]. Additionally, spatial compartmentalization employs BBB-penetrant nanoplatforms (e.g., transferrin receptor-functionalized siRNA lipid nanoparticles) for tumor-selective PHGDH silencing in CNS malignancies [95], or systemically restricted inhibitors (e.g., D8 derivatives) that spare neuronal serine biosynthesis [59] (Fig. 4).

### Stemness-differentiation equilibrium and therapeutic navigation

PHGDH bidirectionally governs the CSC equilibrium through context-dependent mechanisms. It enforces stemness maintenance by sustaining redox homeostasis and nucleotide biosynthesis in glioblastoma [32], facilitating ATP-P2X7 signaling in acute myeloid leukemia [67], modulating H3K36me3 epigenetic marks in breast cancer [23], and transcriptionally upregulating pluripotency factors (OCT4, SOX2, KLF4) in thyroid malignancies [63]. Conversely, PHGDH ablation sensitizes NSCLC CSCs to gemcitabine via disrupted self-renewal pathways [33].

Resolution of this paradox necessitates integration of three determinants: CSC differentiation status dictating compartmentalized metabolic dependencies [96], dynamic microenvironmental metabolite availability (e.g., hypoxia-induced SHMT2 rewiring) [55], and SAM-mediated epigenetic plasticity sculpting histone modifications to reinforce stemness or license differentiation [44]. This informs a precision therapeutic paradigm where spatially resolved metabolomics maps CSC-niche nutrient gradients, while timed combinatorial regimens (e.g., PHGDH inhibitors with retinoids) exploit CSC plasticity. Crucially, titration to thresholds that disrupt self-renewal while permitting differentiation commitment transforms PHGDH’s duality into a targetable vulnerability (Fig. 4).

##  Future direction in PHGDH biology and therapeutics

### Spatial metabolic cartography for precision targeting

Resolving PHGDH's contextual paradoxes requires spatial multi-omics platforms that decode tumor micro-compartmentalization at subcellular resolution. Integration of mass spectrometry imaging (MSI) with single-cell transcriptomics and magnetic resonance spectroscopy (MRS) [19, 97] enables 3D metabolic atlases revealing PHGDH activity gradients across vascular, immune, and hypoxic niches [11, 13]. Such approaches dissect microenvironmental crosstalk driving resistance. Examples include cystathionine accumulation in 1p/19q-codeleted gliomas indicating compartmentalized serine flux [97], and PHGDH-mediated vascular dysregulation underscoring stroma-tumor metabolic interdependence [13]. Advanced modalities like nanoparticle-enhanced SERS imaging could dynamically map metabolic flux within immune-excluded niches [98], elucidating spatial determinants of therapeutic response [53].

Critical research priorities include spatiotemporal tracking of PHGDH nuclear translocation at tumor-nerve interfaces to decode perineural invasion mechanisms [11], and single-cell metabolomic profiling of PHGDH^high^ populations in immunosuppressive niches [19]. These efforts will enable spatially precise interventions (e.g., acidosis-activated prodrugs) that transform paradoxical biology into targetable vulnerabilities.

### Dynamic metabolic biosensing

Real-time biosensing technologies represent the next frontier for monitoring therapeutic adaptation. Genetically encoded reporters visualize serine flux rewiring under glucose deprivation [99] and post-inhibition compensatory pathways [92]. Clinical translation requires "metabolic liquid biopsy" platforms adapting epitranscriptomic detection principles (e.g., GLORI-seq [37]) for continuous serum serine monitoring. Multimodal integration with nanoparticle-SERS [98] permits dynamic mapping of metabolic adaptations in patient-derived xenograft models.

Implementation requires optimization of biosensors for clinical biofluid analysis, correlation of serine flux oscillations with tumor progression metrics, and co-development of theragnostic systems coupling biosensors with adaptive dosing regimens. Such platforms preempt resistance by decoding context-dependent vulnerabilities.

### AI-accelerated therapeutic innovation

Artificial intelligence overcomes limitations of conventional inhibitors through generative modeling of cryptic allosteric sites (e.g., K289 acetylation pocket [18, 58]) and simulation of pathogenic interactomes (e.g., PHGDH-METTL3 [64]) for targeted disruption. This paradigm shift enables PROTACs exploiting tissue-specific vulnerabilities (e.g., PHGDH-interactome interface [58]), and in silico clinical trials using organoid flux data to predict efficacy/resistance.

### Precision clinical translation framework

PHGDH-targeted therapies demand a paradigm shift from histology-driven to biomarker-guided "metabolocentric" trials. We propose a three-pillar framework integrating biomarker stratification, adaptive trial designs, and neurotoxicity-mitigated dosing strategies.

#### Biomarker-stratified patient selection

Robust patient selection for PHGDH-directed therapies requires validated biomarker thresholds. PHGDH IHC H-score ≥ 200 predicts SSP dependency in TNBC/PDAC (ORR increase 4.2-fold) [82, 83]. Parkin deficiency (IHC 0/1 +) stratifies NCT-503-sensitive breast cancer (78% PPV). m^6^A signatures (RNA-seq peak score > 4.0 at *PHGDH* 3'-UTR) indicate METTL3/IGF2BP3-driven resistance [37]. 11q deletion/MYCN amplification co-occurrence in neuroblastoma (HR = 3.1 for response) [100]. Validation progresses through retrospective thresholds, prospective biomarker-embedded trials (e.g., PHOENIX study), and CLIA-certified assay deployment.

##### Neurotoxicity-mitigated therapeutic schema

Compartmentalized therapeutic delivery stratifies administration based on tumor localization. For peripheral malignancies, BBB-sparing inhibitors (e.g., D8 derivatives; PS < 5 × 10⁻^6^ cm/s [59]) are administered at 10 mg/kg twice weekly alongside concomitant L-serine neuroprotective supplementation (300 mg/kg/day) [36]. Dosing should maintain plasma serine concentrations > 100 μM to ensure neuronal safety [36].Conversely, CNS tumors necessitate BBB-penetrant nanoplatforms exemplified by transferrin receptor-functionalized siRNA-loaded lipid nanoparticles, which achieve ≥ fivefold tumor-to-normal tissue delivery ratios [95] administered at 1.5 mg/kg weekly across four treatment cycles.

Neurotoxicity mitigation employs a chronotherapeutic approach integrating weekday inhibitor dosing with weekend L-serine boluses (500 mg/kg) [95], while neuronal integrity is monitored through cerebrospinal fluid (CSF) serine quantification (> 30 μM via LC–MS/MS) [94].

##### PHOENIX basket trial 2.0 design

This biomarker foundation informs the PHOENIX trial (PHGDH Oncology Exploitation via Niche-Integrated eXperimentation), a "basket 2.0" design with three biomarker-stratified arms.

Arm A enrolls PHGDH^CNV+^ solid tumors to target brain metastases by exploiting BBB serine scarcity (PFS endpoint) [95].

Arm B focuses on ATF4^high^/PHGDH^+^ hematologic malignancies to leverage ER-stress vulnerability (CR rate + neurotoxicity) [10, 30].

Arm C selects m^6^A^sig+^/PHGDH^+^ tumors to counteract RNA stabilization-driven resistance (ORR + metabolic escape) [37].

Adaptive features include interim PET/MRS at Week 4 (≥ 30% reduction of ^18^F-serine uptake triggers combo therapy e.g., + NAMPTi [54]) and real-time serum serine monitoring via Liquid Chromatography-Mass Spectrometry (LC–MS/MS) (> 100 μM mandates L-serine rescue [36]).

This integrated blueprint transforms paradoxes of PHGDH into clinically actionable vulnerabilities through spatial biology, dynamic monitoring, computational drug discovery, and biomarker-driven trial design.

While the aforementioned precision framework provides a roadmap for translating PHGDH biology into clinical trials, its successful implementation hinges on resolving fundamental paradoxes and unanswered questions that permeate PHGDH research. These challenges, which span from basic biology to therapeutic application, define the critical frontiers of the field. They are summarized in Table 2 and form the basis for the ongoing evolution of PHGDH-targeted strategies.

**Table 2:** Unresolved questions and future frontiers in PHGDH biology and targeting

Unresolved ouestion/controversy	Key challenges	Proposed experimental approaches	Refs.
The Metastatic Paradox	How to target the anabolic functions of PHGDH inprimary tumors without selecting for pre-existing,invasive PHGDH-low clones?	Pre-treatment assessment of PHGDH/HEXB heterogeneity in CTCs Neoadjuvant models testing PHGDHi + anti-sialylation agent combinations	[15]
Neurotoxicity vs. CNS Efficacy	Can we achieve therapeutic PHGDH inhibition inbrain metastases or glioblastoma without causingneuronal demyelination?	Advanced delivery systems (BBB-penetrantnanoparticles, PROTACs) Chronotherapeutic dosing with L-serinerescue	[36, 95]
Non-Canonical vs. Catalytic Functions	Are the moonlighting functions of PHGDHindependent of its metabolic activity? Can they beselectively targeted?	Structural studies of nuclear/mitochondrial PHGDH complexes Development of reagents that disrupt specificprotein interactions (e.g., PHGDH-METTL3)	[64]
Predictive Biomarkers	Beyond PHGDH mRNA/protein levels, whatfunctional readouts (e.g., PTMs, flux rates) reliablypredict dependency?	Spatial metabolomics to measure in situ pathwayactivity Proteomic profiling of activating PTMs (e.g., R-methylation) in patient samples	[19, 20, 46]
immunomodulatory Roles	What is the net effect of PHGDH inhibition on thetumor immune landscape, given its roles in bothcancer and immune cells?	Single-cell RNA-seq/metabolomics of TME inPHGDHi-treated models Conditional knockout models to dissect cell-autonomous vs.non-autonomous effects	[13, 69]

### Resolving therapeutic resistance paradoxes

The efficacy of PHGDH-targeted therapies is confronted by two distinct, yet potentially co-occurring, resistance paradigms that demand divergent interception strategies.

Adaptive rewiring rapidly induces SLC1A4-mediated serine uptake via ATF4 activation [10]. It is addressed by frontline PHGDHi + SLC1A4i combinations [36] monitored via ^18^F-based PET for metabolic imaging [21].

Pre-existing PHGDH-low clones exploit αvβ₃-integrin hypersialylation (HEXA-HEXB axis) [15]. They are intercepted by neoadjuvant PHGDHi or consolidation anti-sialylation agents (ST6GAL1/ST3GAL4 inhibitors) [15] and pre-treatment dietary serine/glycine restriction to reduce PHGDH-low fitness [36, 75]. Monitoring uses CTC-based assessment of PHGDH/HEXB expression [15].

Emerging mechanisms (SF3B1 spliceosome mutations [79], endothelial hypervascularization [13]) require precision stratification via resistance biomarkers (CTC PHGDH/HEXB, SF3B1) within trials like PHOENIX Arm C.

## Resolving contextual determinants of PHGDH dependency and therapeutic paradoxes

The extensive body of research summarized herein reveals that PHGDH is not a universal oncoprotein but a context-dependent rheostat of tumor fitness. Its dependency and functional output are dictated by a hierarchy of determinants that vary across cancer types (Summarized in Table 3). A critical synthesis of these factors is essential to reconcile paradoxical findings and guide precision therapeutic development.

**Table 3:** Determinants of PHGDH Dependency Across Cancer Lineages

Cancer type	Key oncogenic driver/context	PHGDH role and dependency	Mechanistic basis	Therapeutic lmplication
Ewing sarcoma	EWS-FLl1 fusion	High Dependency	Direct transcriptional activation of SSP genes by fusion oncoprotein [5.24]	Sensitive to direct PHGDH inhibitors (e.g.. NcT-503)[31]
Neuroblastoma	MYC/MYCN amplification	High Dependency	MYC-driven transcriptional program enforces ae novo serine synthesis for nucleotide production [4]	PHGDH inhibition is cytostatic.resistance emerges wa rewinng[4]
Triple-Negative BreastCancer (TNBC)	Genomic Amplification/Basal Context	High Dependency	Sustains nucleotide synthesis, redox balance.and feeds PRMT1-FASN chemoresistance axis[20]	Co-targeting PHGDH or its upstream molecules and FASN(e.g.. TVB-2640) is synergistic[20]
Luminal Breast Cancer	Serine Auxotrophy	Contextual vuinerability	PSAT1 silencing creates dependency one xogenous serine: SSP is less active[88]	Dietary serine restriction may be particularly effective[88]
Glioblastoma	Hypoxia, ER Stress	Stress-Induced Dependency	Upregulated by ATF4/ERNl axis under hypoxia/ER stress: essential for redox balance in GsCs[29, 30]	Potentiates radio/CAR-T therapy but poses neurotoxicity challenge[13, 16]

First, lineage-specific oncogenic drivers create a transcriptional imperative for SSP activation. For instance, in Ewing sarcoma, the EWS-FLI1 fusion oncoprotein directly occupies the *PHGDH* promoter, making SSP flux a cornerstone of its metabolic program [5, 24]. Similarly, MYC-family amplifications in medulloblastoma and neuroblastoma enforce a high-flux serine synthesis state to support nucleotide production [3, 4]. In contrast, cancers without such strong transcriptional drivers may exhibit more flexible, microenvironment-responsive PHGDH expression.

Second, the tissue of origin and its inherent metabolite availability fundamentally shape dependency. This is starkly illustrated in breast cancer subtypes. Luminal subtypes often silence *PSAT1*, rendering them serine auxotrophic and dependent on exogenous serine [88]. Conversely, basal-like/TNBCs frequently amplify or overexpress PHGDH, often associated with the CK5-positive cell lineage, establishing a non-auxotrophic state that supports their aggressive phenotype [83, 84]. Thus, the same metabolic pathway confers vulnerability in one context and dependency in another, based on the baseline metabolic architecture.

Third, the paradoxical role of PHGDH in metastasis is resolved by considering spatial metabolic constraints. In nutrient-replete primary sites and established metastatic niches (e.g., bone, brain), high PHGDH activity fuels anabolic growth and antioxidant defense [2, 50, 87]. However, during the stresses of dissemination and circulatory transit, PHGDH-low clones can exhibit a fitness advantage. They reduce anabolic burden and exploit alternative metabolic pathways such as αvβ₃-integrin hypersialylation to enhance survival and invasion [15]. This suggests that therapeutic inhibition must be carefully timed and combined with anti-integrin strategies to avoid selecting for these pre-adapted, invasive clones.

Finally, the tissue-specific PTM landscape and protein interactome fine-tune PHGDH function, creating distinct therapeutic vulnerabilities. For example, in TNBC, a self-reinforcing PRMT1-PHGDH-FASN axis drives chemoresistance, making this subset uniquely susceptible to FASN inhibition [20]. In HCC, PHGDH's interaction with KIF15 stabilizes the enzyme to maintain stemness [48], while in other contexts, ASS1 binding promotes its degradation [47]. This indicates that effective targeting will require not just inhibiting PHGDH's catalytic activity, but also disrupting its pathogenic protein–protein interactions in a lineage-aware manner.

##  Conclusion

PHGDH exemplifies the complex interplay between metabolic reprogramming, oncogenic signaling, and therapeutic vulnerability in cancer biology. As this review delineates, PHGDH transcends its canonical role in serine biosynthesis to function as a central stress integrator and signaling nexus, dynamically regulated through multilayered epigenetic, transcriptional, post-transcriptional, and post-translational mechanisms [7–9, 20, 23]. Pathological PHGDH overexpression is orchestrated by lineage-specific oncogenic drivers (e.g., EWS-FLI1 in Ewing sarcoma [5], MYC in medulloblastoma [3]), stress-responsive transcription factors (e.g., ATF4 under nutrient deprivation [9, 10]), and RNA epitranscriptomic modifications (e.g., m^6^A/IGF2BP3 stabilization [37]). PTMs further refine its activity: monoubiquitination by Cul4A-DDB1 enhances SAM production for epigenetic metastasis programming [44], while PRMT1-mediated methylation activates a chemoresistant PHGDH-FASN axis in TNBC [20]. These targetable vulnerabilities are counterbalanced by compensatory adaptations, such as mTORC1-driven lipid remodeling [14], underscoring the imperative for rational combinatorial strategies.

Metastatic organotropism reveals functional dichotomies. PHGDH promotes brain metastasis by overcoming BBB serine scarcity [17], yet its inhibition may select for invasive PHGDH-low clones in CRC [15]. Similarly, PHGDH sustains cancer stemness in glioblastoma [32] and AML [67], while its ablation triggers differentiation in NSCLC [33]. This highlights its role as a rheostat of cellular plasticity [96]. The therapeutic promise of PHGDH inhibition is tempered by neurotoxicity risks stemming from central serine depletion [16, 101], recapitulating neurological deficits observed in Neu-Laxova syndrome [93]. Mitigation requires compartmentalized approaches such as BBB-sparing strategies (e.g., D8 derivatives [59], siRNA nanocarriers [95]) or adjunctive L-serine supplementation [102].

Ultimately, PHGDH embodies three cardinal paradoxes of cancer metabolism: a metabolic lifeline under stress that becomes a therapeutic liability; a promoter of primary tumorigenesis that may suppress metastasis; and an enzymatic workhorse moonlighting as an epigenetic regulator. Resolving these contradictions demands precision metabolic oncology by integrating spatial multi-omics, dynamic biosensing, and AI-driven therapeutic reengineering to transform PHGDH from a biological enigma into a clinically tractable target. As context-dependent regulatory rules are deciphered, PHGDH-targeted therapies stand poised to redefine cancer treatment, one metabolic niche at a time.

## Data Availability

No datasets were generated or analysed during the current study.
